# KNOTTED1-LIKE HOMEOBOX 3: a new regulator of symbiotic nodule development

**DOI:** 10.1093/jxb/erv414

**Published:** 2015-09-07

**Authors:** M. Azarakhsh, A. N. Kirienko, V. A. Zhukov, M. A. Lebedeva, E. A. Dolgikh, L. A. Lutova

**Affiliations:** ^1^Department of Genetics and Biotechnology, St Petersburg State University, 199034, St Petersburg, Russia; ^2^All-Russia Research Institute for Agricultural Microbiology (ARRIAM), 196608, St Petersburg, Russia

**Keywords:** Cytokinin, KNOX transcription factors, KNOX3, legume–rhizobium symbiosis, nodule development, plant meristem.

## Abstract

KNOX transcription factors (TFs) regulate different aspects of plant development essentially through their effects on phytohormone metabolism. In particular, KNOX TF SHOOTMERISTEMLESS activates the cytokinin biosynthesis *ISOPENTENYL TRANSFERASE* (*IPT*) genes in the shoot apical meristem. However, the role of KNOX TFs in symbiotic nodule development and their possible effects on phytohormone metabolism during nodulation have not been studied to date. Cytokinin is a well-known regulator of nodule development, playing the key role in the regulation of cell division during nodule primordium formation. Recently, the activation of *IPT* genes was shown to take place during nodulation. Therefore, it was hypothesized that KNOX TFs may regulate nodule development and activate cytokinin biosynthesis upon nodulation. This study analysed the expression of different *KNOX* genes in *Medicago truncatula* Gaertn. and *Pisum sativum* L. Among them, the *KNOX3* gene was upregulated in response to rhizobial inoculation in both species. pKNOX3::GUS activity was observed in developing nodule primordium. *KNOX3* ectopic expression caused the formation of nodule-like structures on transgenic root without bacterial inoculation, a phenotype similar to one described previously for legumes with constitutive activation of the cytokinin receptor. Furthermore, in transgenic roots with *MtKNOX3* knockdown, downregulation of A-type cytokinin response genes was found, as well as the *MtIPT3* and *LONELYGUY2* (*MtLOG2*) gene being involved in cytokinin activation. Taken together, these findings suggest that *KNOX3* gene is involved in symbiotic nodule development and may regulate cytokinin biosynthesis/activation upon nodule development in legume plants.

## Highlight

*KNOX3* gene is involved in symbiotic nodule development in *Medicago truncatula* and *Pisum sativum*, providing evidence that it may regulate cytokinin biosynthesis/activation upon nodulation.

## Introduction

Interaction between legume plants and soil rhizobia leads to the development of new organs on roots, symbiotic nodules, where nitrogen fixation takes place. Bacterial signals Nod factors induce a complex of responses in epidermal cells and stimulate cell proliferation in the root pericycle and cortex, leading to nodule primordium formation (Schultze and Kondorosi, 1998). In legumes with indeterminate nodules, such as *Medicago truncatula* Gaertn. and *Pisum sativum* L., the nodule meristem is formed at the tip of primordium, providing the indeterminate growth of nodules (Rolfe and Gresshoff, 1988; Sprent and James, 2008).

A large number of studies on the model legumes have been carried out, demonstrating the important role of cytokinin and auxin in regulation of cell proliferation and differentiation during nodule development ((Libbenga *et al.* 1973; Gonzalez-Rizzo *et al.*, 2006; Tirichine *et al.*, 2007; Heckmann *et al.*, 2011; Rightmyer and Long, 2011; Plet *et al.*, 2011, reviewed by Ferguson and Mathesius, 2014). The positive role of cytokinin in nodule formation was first demonstrated in experiments with exogenous application of cytokinin, which promotes the formation of nodule-like structures on the roots of legume plants (Libbenga *et al.* 1973, Cooper and Long, 1994; Heckmann *et al.*, 2011). Furthermore, a gain-of-function mutation in the *Lotus histidine kinase1* (*LjLHK1*) gene encoding the cytokinin receptor in *Lotus japonicus* leads to spontaneous nodule formation (Murray *et al.* 2007; Tirichine *et al.*, 2007). The *lhk1-1* mutant carrying a loss-of-function mutation in the cytokinin receptor gene has reduced nodule formation, but it still develops a limited number of nodules at a later time point after rhizobial inoculation (Murray *et al.*, 2007). Recently, it was shown that, along with LjLHK1, other cytokinin receptors such as LjLHK1A and LjLHK3 work partially redundantly promoting cell divisions during nodule primordium formation in *L. japonicus* (Held *et al.*, 2014). Cytokinin-responsive genes, type-A response regulators, are activated in response to rhizobial inoculation, indicating that the cytokinin response is a part of a signalling cascade induced by rhizobia (Gonzalez-Rizzo *et al.*, 2006; Op den Camp *et al.*, 2011; Plet *et al.*, 2011). In agreement with this, cytokinin activates the expression of genes encoding transcriptional regulators of nodule development, such as NSP1, NSP2, ERN1, and NIN (Plet *et al.*, 2011). Recently, it was shown that the expression levels of genes involved in cytokinin biosynthesis were increased early in response to rhizobial signals. In *L. japonicas*, the expression level of the *LjIPT3* gene encoding adenylate isopenthenyltransferase, the first and rate-limiting enzyme in cytokinin biosynthesis, was induced within 3h after *Rhizobium* inoculation. Its expression increased in roots during nodule development, reaching the highest level at the mature stage. Moreover, the expression of *LjIPT1* was shown to be increased at 7 d after inoculation (Chen *et al.*, 2013). In *M. truncatula*, the expression of *MtIPT* (*Medtr2g022140*) was induced as early as 1h after Nod factor treatment (van Zeijl *et al.*, 2015). In addition, *LONELYGUY* (*LOG*) genes encoding cytokinin-activating enzymes were upregulated during nodulation in *Medicago* (Mortier *et al.*, 2014, van Zeijl *et al.*, 2015). Nod factor treatment was shown to induce accumulation of cytokinins, including *trans*-zeatin and isopentenyl adenine, in the root susceptible zone within 3h (van Zeijl *et al.*, 2015). Taken together, these data suggest that cytokinin biosynthesis and activation are induced in legume roots in response to rhizobial signals.

Recently, it was shown that the cytokinin biosynthesis genes *LjIPT3* and *LjIPT1* were also induced in the shoot after rhizobial inoculation. Furthermore, *LjIPT3* was shown to be activated in shoot phloem via the components of the AON (autoregulation of nodulation) system, thereby negatively affecting nodulation. Nodule number was increased in mutants defected in *LjIPT3* and decreased in *LjIPT3*-overexpressing plants (Sasaki *et al.*, 2014). In contrast to this, in previous study, Chen *et al.* (2013) found that RNA interference (RNAi) knockdown lines of *LjIPT3* had decrease nodule number, suggesting that *LjIPT3* has a positive effect on nodule development. Therefore, it was proposed that cytokinins have a dual role in nodulation depending on the time and place of their induction (Sasaki *et al.*, 2014).

Along with cytokinin, auxin is also involved in nodule development. Exogenous application of polar auxin transport inhibitors stimulated the formation of nodule-like structures (Hirsch *et al.*, 1989). There is evidence that polar auxin transport is regulated by cytokinin, as cytokinin negatively regulates the expression of *PIN* genes encoding auxin efflux carriers that mediate polar auxin transport (Plet *et al.*, 2011).

The role of cytokinin and auxin as well as their crosstalk is well studied for plant meristems, specifically shoot and root apical meristems (SAM and RAM, respectively). In plant meristems, phytohormone metabolism, their transport and crosstalk are regulated by a set of transcription factors (TFs). A special place among them belongs to the TFs with a homeodomain of the WUSCHEL-RELATED HOMEOBOX (WOX) and KNOTTED-LIKE HOMEOBOX (KNOX) families (Mayer *et al.*, 1998; Schoof *et al.*, 2000; Hake *et al.*, 2004; Sarkar *et al.*, 2007).

Little is known about the role of meristem-specific TFs in nodulation. Previously, the TF WOX5, a member of the WOX family, was shown to be involved in nodule development (Osipova *et al.*, 2012). However, the role of *KNOX* genes in the development of symbiotic nodules in legume plants remains unexplored.

The best-characterized member of the KNOX family, SHOOTMERISTEMLESS (STM), stimulates the expression of *IPT* genes in the SAM. Based on sequence similarity, gene structure, and expression pattern, KNOX TFs are grouped into two classes, class I KNOX (KNOXI) and class II KNOX (KNOXII) (Hay and Tsiantis, 2010). *KNOXI* genes, which are evolutionarily close to KNOTTED1 from maize, the first homeodomain-containing TF identified in plants (Vollbrecht *et al.*, 1991), are expressed in the SAM and play crucial roles in the maintenance of the SAM (Long *et al.*, 1996; Hake *et al.*, 2004; Barkoulas *et al.*, 2008; Hay and Tsiantis, 2010). *KNOXII* genes display diverse expression patterns and their function is not yet well understood (Serikawa *et al.*, 1997; Byrne *et al.*, 2002; Zhong *et al.*, 2008).

*KNOX* genes have been characterized in legume plants. In total, 10 *KNOX* genes have been identified in *M. truncatula* (Di Giacomo *et al.*, 2008, Peng *et al.*, 2011; Zhou *et al.*, 2014). In pea (*P. sativum* L.), two members of *KNOX1* class, the *KNOX1* and *KNOX2* genes, have mainly been investigated and showed to be involved in the regulation of plant architecture (Hofer *et al.*, 2001; Zhou *et al.*, 2014).

This study questioned whether *KNOX* genes regulate nodule formation in legumes and whether they may regulate the expression of cytokinin biosynthesis genes upon nodulation in a manner similar to their action in the SAM. It was found that the *KNOX3* gene was upregulated in response to rhizobial inoculation, and its promoter activity was observed in developing nodule primordium. Data from *KNOX3* ectopic expression and RNAi suggested that the *KNOX3* gene may regulate cytokinin biosynthesis and activation during nodule development.

## Materials and methods

### Plant material, bacterial strains, and growth conditions

*M. truncatula* Gaertn. Jemalong plants (wild-type A17) were grown in growth chambers (16h/8h day/night regime, 21 °C, and 75% relative humidity) and inoculated with *Sinorhizobium meliloti* strain Sm2011. The seeds were surface sterilized with concentrated sulphuric acid for 10min and washed five to six times with sterile water. *Medicago* seedlings were inoculated with 1ml of culture of per plant (OD_600_=0.7). For temporal expression during nodulation, *M. truncatula* plants inoculated with Sm2011 were grown in vermiculate-containing pots moistened with nitrogen-free Farhaeus medium (Fahraeus, 1957), and infected root tissue was harvested at different stages after inoculation with *S. meliloti* together with the uninoculated control plants. To avoid harvesting of lateral root primordia, only segments between emerged lateral roots were collected. Nodules were obtained at different stages after inoculation from the infected sites of roots.

*P. sativum* L. cv. Frisson seeds were surface sterilized with sulphuric acid for 5min, washed three times with water, transferred to 1% water agar plates and germinated at room temperature in the dark. After germination (for 4–5 d), plants were transferred into pots with vermiculite saturated with Jensen medium (van Brussel *et al.*, 1982) and grown in a growth chamber at 21 °C in a 16h/8h light/dark cycle at 60% humidity. Pea seedlings were inoculated with 1ml of culture of *Rhizobium leguminosarum* bv. *viciae* CIAM1026 (ARRIAM, WDCM 966) per plant (OD_600_=0.5), and infected root tissue was harvested at different stages after inoculation together with the uninoculated control plants..

### Molecular cloning

The *MtKNOX3* promoter (2204bp) and the coding sequence of *MtKNOX3* were first cloned into pDONR221 (Invitrogen, USA) and then into pBGFWS7.0 containing the β-glucuronidase (*GUS*) reporter gene and 35S terminator sequence and the pB7WG2D vector containing the 35S promoter and terminator sequences (VIB, Ghent, Belgium), respectively, with LR clonase enzyme (Invitrogen). For downregulation of the *MtKNOX3* gene, a fragment of nt 1–151 in the coding sequence was amplified with the addition of the CACC sequence to the forward primer and cloned in the pENTR-D-TOPO vector (Invitrogen) and was cloned in the pK7GWIWG2D vector using LR clonase enzyme. The resulting vector contained a hairpin construct flanked by the 35S promoter and terminator sequences. The primers used for cloning are listed in Supplementary Table S1, available at *JXB* online. All the cloning vectors were sequenced.

The full-length coding sequence of the *PsKNOX3* gene was amplified using cDNA as a matrix with corresponding primers (Supplementary Table S1). Amplification was done using Phusion Flash High-Fidelity PCR Master Mix (Thermo Scientific, USA). The amplified product was inserted in the pDONR221 vector with BP Clonase enzyme (Invitrogen) and finally into pB7WG2D with the LR clonase enzyme. The verified construct was transferred into *Agrobacterium rhizogenes* strain Arqua 1. The pB7WG2D and pK7GWIWG2D vectors used in this work contain the green fluorescent protein (GFP) marker gene for the selection of transgenic roots.

To identify putative *KNOX* genes in *P. sativum*, a BLASTN search was performed on the recently created Transcriptome Shotgun Assembly (TSA) [roots of pea line SGE inoculated with *R. leguminosarum* bv. *viciae* at 7 d post-inoculation (dpi); V. Zhukov, A. Zhernakov, O. Kulaeva, N. Ershov, A. Borisov and I. Tikhonovich, unpublished data) and other available TSAs of *P. sativum* L. [BioProject: PRJNA66035 (Franssen *et al.*, 2011), BioProject: PRJNA81209 (Kaur *et al.*, 2012), BioProject: PRJNA211622 (Duarte *et al.*, 2014)] using full-length *KNOX1–KNOX10* cDNAs of *M. truncatula*. This allowed the identification of five predicted full-length sequences that showed a high level of homology to *MtKNOX3*, *MtKNOX4*, *MtKNOX5*, *MtKNOX9*, and *MtKNOX10*, and these were named *PsKNOX3*, *PsKNOX4*, *PsKNOX5*, *PsKNOX9*, and *PsKNOX10.* The full-length coding sequences of *PsKNOX3*, *PsKNOX4*, *PsKNOX5*, *PsKNOX9*, and *PsKNOX10* genes were verified by PCR amplification on cDNAs (synthesis was done on total RNA isolated from inoculated pea roots of the SGE line at 7 dpi) using the primer pairs listed in Supplementary Table S1, available at *JXB* online, and flanking predicted coding sequences.

In addition, partial transcripts of four predicted pea *KNOX* genes were identified and named *PsKNOX1*, *PsKNOX2*, *PsKNOX6*, and *PsKNOX8*. The RACE procedure was used to reveal the full-length coding sequences of the *PsKNOX6* and *PsKNOX8* genes using the primer pairs listed in Supplementary Table S1. Synthesis was done on total RNA isolated from inoculated pea roots of the SGE line for *PsKNOX8* gene, but for *PsKNOX6* total RNA was isolated from leaves, because *PsKNOX6* was barely expressed in pea roots and nodules.

The full-length sequence of the *PsKNOX3* gene was also identified by RACE on cDNA of *P. sativum* cv. Frisson as a template (Supplementary Table S1). Since experimental work was done on *P. sativum* cv. Frisson, the specificity of the designed primers for PCR and sequenced all amplicons was checked.

### Quantitative reverse transcription PCR (qRT-PCR) analysis

Total RNA was isolated from roots with an RNeasy Plant Mini Kit (Qiagen, Germany) according to the manufacturer’s instructions. After a DNase (Thermo Scientific) treatment, the samples were extracted with an equal volume of chloroform, and RNA was precipitated from the aqueous phase with 3M sodium acetate and ethanol and subsequently quantified with a NanoDrop 2000c UV-Vvis Spectrophotometer (Thermo Scientific). RNA (from 200ng to 1 μg) was used for cDNA synthesis with RevertAid Reverse Transcriptase (Thermo Scientific). The efficacy of the DNase treatment was checked by using controls without reverse transcriptase for subsequent qRT-PCR analysis. The qRT-PCR experiments were done on a CFX-96 real-time PCR detection system with a C1000 thermal cycler (Bio-Rad Laboratories, USA), and SYBR Green and Eva Green intercalating dye were used for detection (Bio-Rad Laboratories). All reactions were done in triplicate and averaged. Cycle threshold (*C*_t_) values were obtained with the accompanying software and data were analysed with the 2^–ΔΔ *C*t^ method (Livak and Schmittgen, 2001). The relative expression was normalized against the constitutively expressed ubiquitin and actin genes in *Medicago* and pea. *Medicago* cDNA sequences were taken from *the M. truncatula* genome database Mt4.0v1. All primer pairs (Supplementary Table S2) were designed using the Vector NTI program and produced by Evrogen (http://www.evrogen.com). PCR amplification specificity was verified using a dissociation curve (55–95 °C). Each experiment was repeated at least three times with independent biological samples.

### *A. rhizogenes*-mediated plant transformation

*A. rhizogenes*-mediated *M. truncatula* plants were transformed as described previously (Mortier *et al.*, 2010). For pea transformation, the seeds were sterilized as described above. Seeds were germinated on 1% water agar in the darkness. Five-d-old seedlings were transferred in sterile plastic bags with Jensen medium to the light and incubated for 2–3 d. The pea roots were cut off in the area of the hypocotyl and transformed with freshly grown *A. rhizogenes* strain Arqua 1. Plants were put in special glass jars 120×60mm on Jensen agar (van Brussel *et al.*, 1982), and the area of cut-off was covered with wet wool and foil. The seedlings were co-cultivated with *A. rhizogenes* for 10–12 d at 21 °C (16h/8h light/darkness) and subsequently transferred to Emergence medium (Limpens *et al.*, 2004) containing 150mg ml^–1^ of cefotaxime. Plants were incubated on Emergence medium with antibiotic for 7 d and then transferred to fresh Emergence medium without antibiotic and incubated for 4–5 d until the new roots were formed (potentially co-transformed with the T-DNA of the binary vector). Emerging roots were analysed using an epifluorescent stereomicroscope (Stereo Discovery V8; Carl Zeiss). The plants were placed into vermiculite and kept under plastic film for 2–3 d. After inoculation with *R. leguminosarum* bv. *viciae* (OD_600_=0.1–0.2), the plants were checked for nodule formation after 14 –21 d.

### Histochemical localization of GUS activity

GUS activity in transformed roots was analysed with 2mM 5-bromo-4-chloro-3-indolyl-β-d-glucuronic acid as substrate in NT buffer (100mm Tris/HCl, pH 7.5, 50mm NaCl) supplemented with 2mM ferricyanide (Van den Eede *et al.*, 1992). The roots were vacuum infiltrated for 20min and subsequently incubated in GUS buffer [100mM Tris, 50mM NaCl, 1.9mM K_3_Fe(CN)_6_, 2.5mM X-Gluc (pre-diluted in DMSO)] at 37° C until sufficient blue staining had been developed (about 1h). After staining, the roots were infiltrated with fixative (3% paraformaldehyde, 0.25 % glutaraldehyde, 0.1 % Tween 20, 0.1 % Triton X-100 in 1/3× MTSB buffer) under vacuum (–1 atm), fixed overnight at 4 °C, and rinsed with 1/3× MTSB buffer (50mm PIPES, 5mm MgSO4, 5mm EGTA). The root segments were subsequently embedded in agarose (3%) and 50 µm sections were prepared with a microtome with a vibrating blade (VT-1200S; Leica). Photographs were taken with a fluorescent microscope AxioImager.Z1 (Carl Zeiss, Germany) equipped with an MRc5 digital camera (Carl Zeiss) and ZEN 2011 microscope software (Carl Zeiss).

### Statistical methods and computer software

Multiple alignment of nucleotide sequences was performed using Clustal W (Thompson *et al.*, 1994) using Vector NTI Advance 10 (InforMax, http://www.informaxinc.com). MEGA6 was used to generate graphic output of phylogenetic tree (Hall, 2013). One-way ANOVA and Student’s *t*-test were used to compare gene expression levels in transgenic roots.

## Results

### qRT-PCR analysis of *KNOX* gene expression upon nodulation in *M. truncatula* and *P. sativum*

Ten members of the *KNOX* family have been identified previously in *M. truncatula* genome (Di Giacomo *et al.*, 2008; Peng *et al.*, 2011; Zhou *et al.*, 2014). In pea, only two genes of this family, *KNOX1* (GenBank accession no. AF080104) and *KNOX2* (AF080105), have been annotated in databases to date (Hofer *et al.*, 2001). To identify other pea *KNOX* genes, the recently created TSA (V. Zhukov, A. Zhernakov, O. Kulaeva, N. Ershov, A. Borisov and I. Tikhonovich, unpublished data) and other available TSAs of *P. sativum* L. (Franssen *et al.*, 2011; Kaur *et al.*, 2012; Duarte *et al.*, 2014) were searched using full-length *KNOX1–KNOX10* cDNAs of *M. truncatula*. As a result, the full-length and partial transcripts of nine predicted pea *KNOX* genes were identified. Two of them, *PsKNOX1* and *PsKNOX2*, corresponded to the previously characterized pea genes. The full-length coding sequences of *PsKNOX* genes were verified by PCR amplification of cDNAs using the primer pairs listed in Supplementary Table S1 and the flanking predicted coding sequences. Subsequent RACE analysis for partial transcripts allowed identification of other full-length pea cDNAs. Finally, identified genes were annotated as *PsKNOX3*–*PsKNOX6*, *PsKNOX8–PsKNOX10* (GenBank accession nos KP296686/KP296687, KP296688, KP296689/KP296690, KT363851, KP296691, and KP296692).

**Fig. 1.**
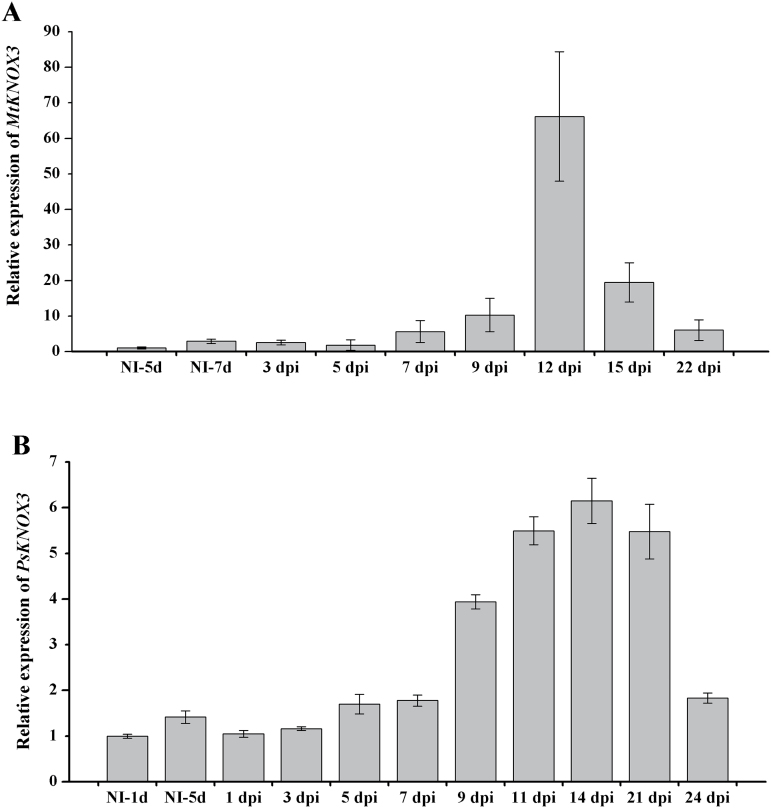
qRT-PCR expression analysis of *MtKNOX3* (A) and *PsKNOX3* (B) in uninoculated plants (NI) and at different dpi. The relative expression was normalized against the constitutively expressed ubiquitin and actin genes. Results are means±SEM of three technical repeats.

To study the expression dynamics of *KNOX* genes during nodule development, the relative transcript levels of *KNOX* genes were analysed at different stages after inoculation in *M. truncatula* and *P. sativum* using qRT-PCR. At 7 dpi, *MtKNOX3* expression increased slightly in roots of *M. truncatula* inoculated with *S. meliloti* in compare with uninoculated control roots (Fig. 1A). At later stages, *MtKNOX3* expression continued to increase up to a maximum at 12 dpi (on average more than 40-fold increase, compared with uninoculated control roots). Later at 15–22 dpi, *MtKNOX3* expression decreased (Fig. 1A). The activation of the *MtKNOX3* gene upon nodule development is consistent with data available in GENEEXPRESSION ATLAS (http://mtgea.noble.org/v3/). In addition to *MtKNOX3*, *MtKNOX5* and *MtKNOX9* were slightly upregulated during nodulation, although not as much as *MtKNOX3* (Supplementary Fig. S1, available at *JXB* online). The expression levels of other *MtKNOX* genes in response to inoculation were not changed significantly (Supplementary Fig. S1).

In accordance with the data obtained for *Medicago*, *PsKNOX3* expression levels significantly increased upon nodulation (Fig. 1B). The level of *PsKNOX3* transcripts increased during nodule development starting from 7 dpi and reached the highest level at mature stages (14–21 dpi). *PsKNOX3* expression then decreased at 24 dpi. At the same time, similarly to *M. truncatula*, the transcriptional levels of *PsKNOX5* and *PsKNOX9* genes were shown to increase upon nodulation (Supplementary Fig. S2, available at *JXB* online). In addition, in pea, the expression of *PsKNOX10* was higher in developing nodules compared with uninoculated control roots. The expression levels of other *PsKNOX* genes did not change significantly in roots after inoculation (Supplementary Fig. S2). Since *MtKNOX3* and its pea orthologue *PsKNOX3* were the most strongly induced following inoculation, the *KNOX3* gene was focused on for further functional studies.

### Tissue-specific expression pattern of *KNOX3* following nodulation by promoter:GUS analysis

**Fig. 2.**
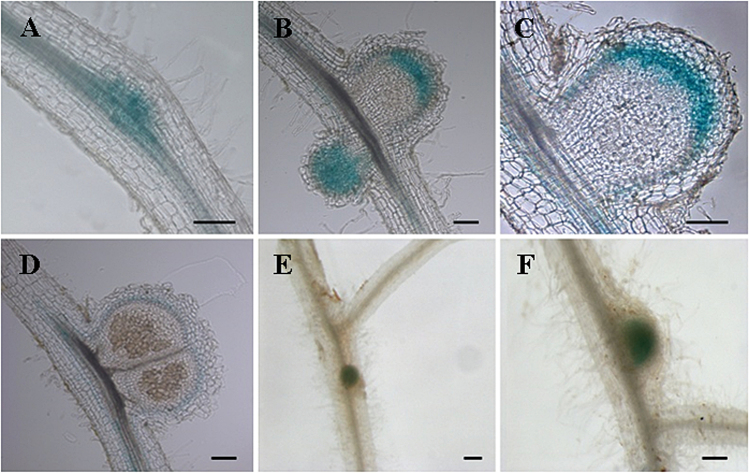
Tissue-specific expression pattern of pMtKNOX3:GUS in *M. truncatula*. (A–D) Bright-field picture of a longitudinal section in nodule primordium (A), throughout proliferating cells in developing nodule (B), in the apical part of the nodule and in provascular bundles (C) and in late stages of nodule development (D). (E, F) pMtKNOX3:GUS expression in developing nodules. Bars, 100 μm. Thickness of sections, 50 μm.

To localize *KNOX3* expression during nodule development, a 2204bp *MtKNOX3* putative promoter region was isolated and used to make a pMtKNOX3:GUS construct (see Materials and methods). This construct was introduced into *M. truncatula* roots via *A. rhizogenes*-mediated transformation. *KNOX3* promoter activity was observed in developing nodule primordia at 7–10 dpi. At later stages (12–15 dpi), pKNOX3:GUS activity was restricted to provascular bundle tissues and to the apical part of nodule where apical meristem is formed (Fig. 2). At 18 dpi, *MtKNOX3* promoter activity was detected at the periphery of mature nodules. The *MtKNOX3* expression dynamic visualized with the *MtKNOX3*:*GUS* fusion was consistent with the qRT-PCR data. In addition to that in developing nodules, *MtKNOX3* promoter activity was also found in lateral root primordia and root tips (Supplementary Fig. S3, available at *JXB* online).

Temporal dynamics of cytokinin response gene expression coincides with *KNOX3* expression during nodulation

**Fig. 3.**
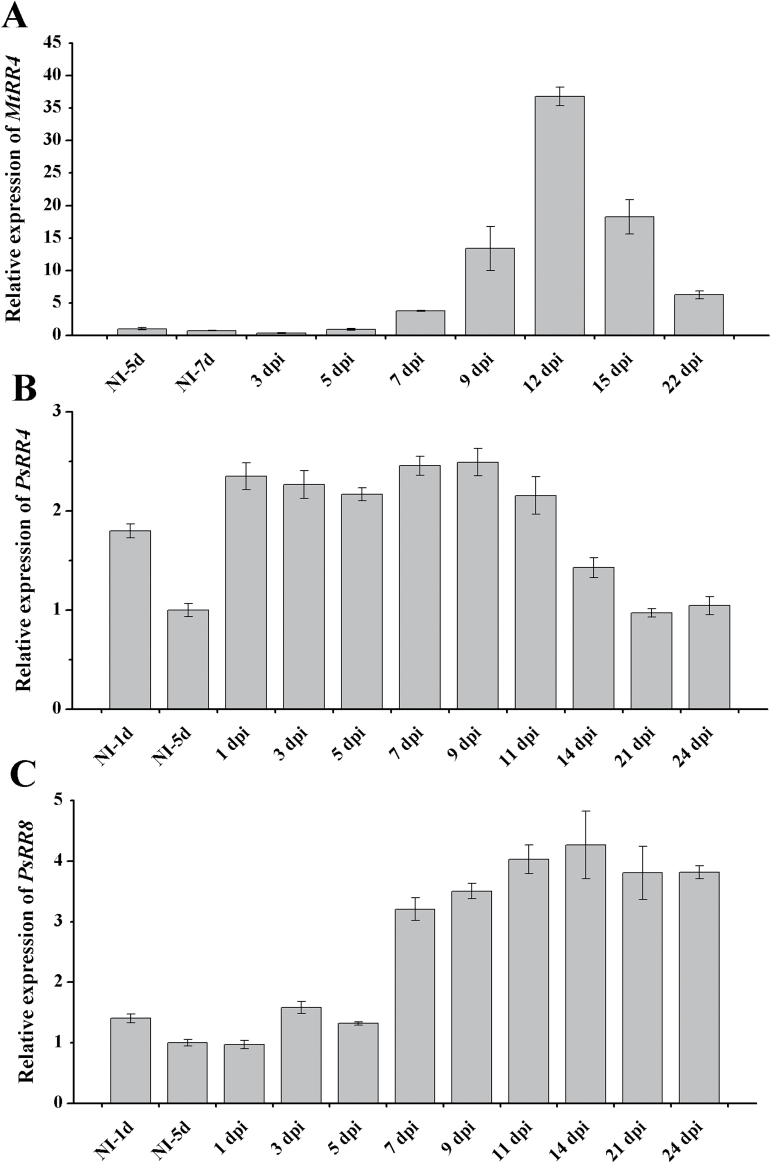
Activation of cytokinin response regulator genes upon nodule development. Expression of *MtRR4* (A) and *PsRR4* (B), and *PsRR8* (C) at the various stages of symbiosis development. NI, not inoculated. The relative expression was normalized against the constitutively expressed ubiquitin and actin genes. Results are means±SEM of three technical repeats.

It is known that cytokinin response genes are activated upon nodule development (Lohar *et al.*, 2004; Tirichine *et al.*, 2007; Plet *et al.*, 2011). It was shown previously that in *M. truncatula* the transcription level of five cytokinin response genes, *MtRR4*, *MtRR5*, *MtRR8*, *MtRR9*, and *MtRR11*, was markedly increased in response to inoculation with rhizobia in a CRE1-dependent manner (Gonzalez-Rizzo *et al.*, 2006; Op den Camp *et al.*, 2011). In our experiments, the expression of cytokinin response gene *MtRR4* in *M. truncatula* reached its maximum at 12 dpi (Fig. 3A), i.e. coinciding with the maximum *MtKNOX3* gene expression observed. In *P. sativum*, the expression of the *PsRR4* (GenBank accession no. KP296699) and *PsRR8* (KP296703) cytokinin response genes (closest homologuws of *MtRR4* and *MtRR8*) were analysed at nodulation, which showed that *PsRR8* expression reached a maximum at 14–21 dpi (Fig. 3B, C). The same expression dynamic was found for *PsKNOX3*. Thus, the *KNOX3* expression maximum in both legumes was associated with the cytokinin response maximum upon nodule development, suggesting that KNOX3 may be involved in the cytokinin response activation.

### Ectopic expression of *KNOX3* causes the formation of nodule-like structures

To investigate the role of KNOX3 in root nodule development, transgenic roots overexpressing *35S:MtKNOX3* and *35S:PsKNOX3* were obtained and analysed by microscopy. qRT-PCR analysis confirmed the ectopic expression of *MtKNOX3* and *PsKNOX3* in individual transgenic roots (data not shown).

**Fig. 4.**
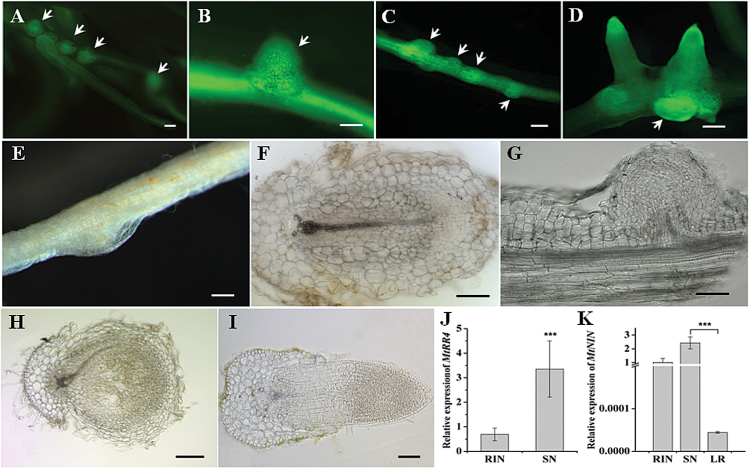
Phenotype of transgenic roots with overexpression of *MtKNOX3* and *PstKNOX3.* The formation of structures resembling nodule primordia (spontaneous nodules) was found in the absence of rhizobia inoculation (A–G). (A–D) GFP fluorescence demonstrated expression of the transformation marker in *M. truncatula* (A, B) and *P. sativum* L. (C, D) Arrows indicate nodule-like structures. (E) Bright-field images of spontaneous nodules in *M. truncatula*. (F, G) Cross-section (F) and longitudinal section (G) of spontaneous nodules in *M. truncatula* with single vascular bundle. (H) Cross-section of wild-type nodule in *M. truncatula* with peripheral vascular bundles. (I) Cross-section of lateral root in *M. truncatula*. (J) Expression of *MtRR4* in nodule-like structures in comparison with rhizobium-induced nodules (RIN) in *M. truncatula*. SN, spontaneous nodule. (K) Expression of nodule-specific genes *MtNIN* in SNs, RINs and lateral roots (LR). Error bars indicate the 95% confidence interval of three biological repeats. Asterisks indicate statistically significant differences compared with control (RIN): ***P<0.001. Bars, 100 μm. Thickness of sections, 50 μm.

In uninoculated *MtKNOX3*-overexpressing and *PsKNOX3*-overexpressing roots, bumps were found resembling root nodule primordia (Fig. 4A–E). Such structures were not observed on the control roots with *35S:GUS* overexpression. The frequency of such nodule-like structure formation was about 10–20 %: seven of 70 *Medicago* plants with GFP-positive transgenic roots formed these bumps (two to three per plant), whereas in pea, three of 15 plants with GFP-positive roots formed the bumps (four to five per plant). Microscopic analysis revealed that these nodule-like structures appeared as a result of cell divisions opposite to the protoxylem pole (Fig. 4F). In contrast to rhizobium-induced nodules, such structures did not develop a peripheral vascular system but instead formed one central vascular bundle similar to the lateral root primordial (Fig. 4F, G). In addition, expression of the nodulation-specific *NIN* gene was tested, which is expressed specifically in nodules and has a significantly lower expression level in lateral roots, as shown previously by Guan *et al.* (2013). *NIN* gene expression was significantly higher in bumps formed on *KNOX3*-overexpressing roots, compared with lateral root primordia. This suggested that the bumps observed were different from lateral root primordia and have nodule-like nature (Fig. 4K).

Spontaneous nodules have been observed previously in legume plants with constitutive activity of cytokinin receptor gene. To check if nodule-like structures on *KNOX3*-overexpressing roots could be developed due to accumulation of cytokinin, the expression level of cytokinin response genes was analysed in these structures. Indeed, in *Medicago*, the expression of *MtRR4* was significantly higher in nodule-like structures compared with rhizobium-induced nodules on *GUS*-overexpressing roots taken at 12 dpi (Fig. 4J).

Since these data supported the hypothesis about the possible role of *KNOX3* gene in activation of cytokinin biosynthesis gene, the next step was to study the expression of cytokinin biosynthesis/activation genes in *KNOX3*-overexpressing and *KNOX3*-RNAi roots.

### Expression of cytokinin biosynthesis/activation genes upon nodule development

To check our hypothesis that KNOX3 TF may activate expression of cytokinin biosynthesis/activation genes, the expression of *IPT* and *LONELY GUY* (*LOG*) genes, encoding cytokinin biosynthesis enzymes isopentenyl transferases and cytokinin-activating enzymes cytokinin riboside 5′-monophosphate phosphoribohydrolases, respectively, was analysed during nodule development in *M. truncatula* and *P. sativum*.

This study focused on the *Medtr2g022140* gene (previously named *MtIPT1* by van Zeijl *et al.*, 2015), which was shown to be induced quite early after Nod factor treatment (1–3h) (van Zeijl *et al.*, 2015), and the *Medicago* homologues of the *LjIPT1* and *LjIPT3* genes, which are induced during nodule development in *L. japonicus* (Chen *et al.*, 2013). The *M. truncatula* genome database Mt4.0v1 was searched using *LjIPT1* and *LjIPT3* as reference sequences and closest homologues of these genes were found in *M. truncatula*. Phylogenetic analysis revealed that the closest homologue of *LjIPT1* was *Medtr1g110590*, and the closest homologue of *LjIPT3* was *Medtr1g072540. Medtr2g022140* appeared to be the closest homologue of the *LjIPT4* gene (Supplementary Fig. S4, available at *JXB* online). To avoid confusion, *Medicago* sequences are referred to according to the numbers given to corresponding *IPT* genes in *L. japonicus*, i.e. *Medtr1g110590* is referred to as *MtIPT1*, *Medtr1g072540* is referred to as *MtIPT3*, and *Medtr2g022140* is referred to as *MtIPT4.*

**Fig. 5.**
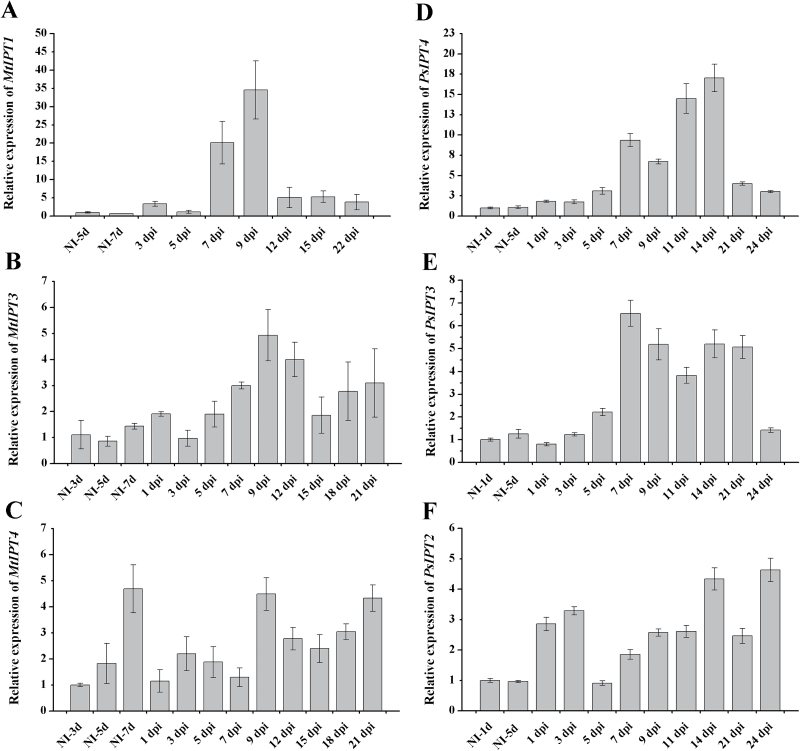
qRT-PCR expression analysis of *MtIPT1* (A), *MtIPT3* (B), *MtIPT4* (C), *PsIPT4* (D), *PsIPT3* (E), and *PsIPT2* (F) genes during nodule development in *M. truncatula* and *P. sativum* L. NI, not inoculated. Quantification was normalized using stable expressed reference genes ubiquitin and actin. Results are means±SEM of three technical repeats.

The expression dynamic of these *MtIPT*s was analysed in response to *Rhizobium* inoculation and it was found that *Medtr1g110590* (*MtIPT1*) gene expression increased dramatically at 7 and 9 dpi (30- and 40-fold, respectively, compared with uninoculated control) (Fig. 5A). Moreover, the expression level of *Medtr1g072540* (*MtIPT3*) was also increased during nodulation (Fig. 5B), whereas *Medtr2g022140* (*MtIPT4*) expression was not changed significantly upon nodule development (Fig. 5C). Therefore, subsequent analyses focused on the *Medtr1g110590* (*MtIPT1*) and *Medtr1g072540* (*MtIPT3*) genes.

In *P. sativum*, two *IPT* genes (previously named *PsIPT1* and *PsIPT2*) have been identified (Tanaka *et al.*, 2006). *PsIPT1* appeared to be the closest homologue of *MtIPT2* (Medtr4g117330) and *LjIPT2*, whereas *PsIPT2* seemed to be the closest homologue of *MtIPT4* (Medtr2g022140) and *LjIPT4* genes (Supplementary Fig. S4). To find *P. sativum* genes corresponding to *M. truncatula* and *L. japonicus IPT1* and *IPT3* genes, an *in silico* search was performed on TSA databases. As a result, two *IPT* genes designated *PsIPT3* (GenBank accession no. KP296693) (the closest homologue of *MtIPT3* (*Medtr1g072540*) and *LjIPT3*) and *PsIPT4* (KP296694) (the closest homologue of *MtIPT1* (*Medtr1g110590*) and *LjIPT1*) were identified.

Similarly to *Medicago*, in pea the transcription level of *PsIPT4* (closest homologue of *MtIPT1* and *LjIPT1*) was shown to be increased significantly during nodulation (Fig. 5D). The level of *PsIPT4* expression increased at 5–7 dpi and reached a maximum at 11–14 dpi (on average a 10- to 15-fold increase compared with uninoculated control roots). The expression of *PsIPT2* gene [closest homologue of *MtIPT4* (*Medtr2g022140*)] was induced at early stages of nodule development (at 1–3 dpi) relative to uninoculated control and was slightly increased at mature stages of nodule development (Fig. 5F). The expression of the *PsIPT3* gene was induced upon nodulation (on average 3- to 4-fold increase) but not as significantly as for *PsIPT4* (Fig. 5E).

**Fig. 6.**
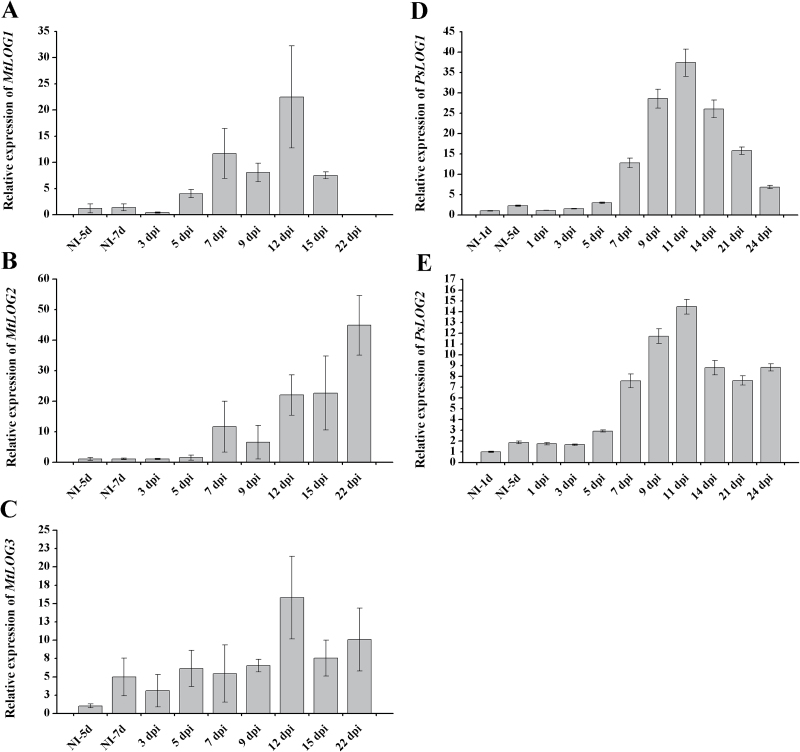
Relative expression of *MtLOG1* (A), *MtLOG2* (B), *MtLOG3* (C), *PsLOG1* (D) and *PsLOG2* (E) genes determined using qRT-PCR during nodule development in *M. truncatula* and *P. sativum* L. NI, not inoculated. The relative expression was normalized against the constitutively expressed ubiquitin and actin genes. Results are means±SEM of three technical repeats.

Among the *MtLOG* genes, *MtLOG1* and *MtLOG2* expression levels were increased upon nodule development (Fig. 6A, B). The highest level of *MtLOG1* transcripts was observed at 12 dpi, whereas *MtLOG2* expression level increased steadily with time after inoculation, reaching the highest level at 22 dpi. The data on *MtLOG1/2* genes expression upon nodule development are consistent with data obtained by Mortier *et al.* (2014). Moreover, the accumulation of *MtLOG3* gene transcripts at 12 dpi (Fig. 6C) was also observed.

Similarly, in *P. sativum*, the closest homologues *PsLOG1* (GenBank accession no. KP296697) and *PsLOG2* (KP296698) showed significant upregulation in roots after rhizobial inoculation. The highest level of *PsLOG1* and *PsLOG2* transcripts was found at 11–14 dpi (Fig. 6D, E).

### Effect of KNOX3 ectopic expression on symbiosis-inducible *IPT* and *LOG* genes

**Fig. 7.**
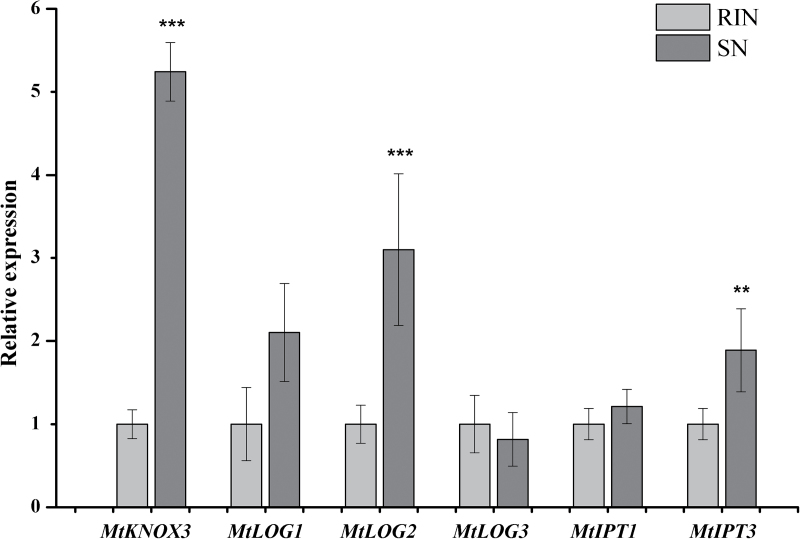
Expression of symbiosis-inducible *IPT* and *LOG* genes in nodule-like structures in comparison with rhizobium-induced nodules (RIN) in *M. truncatula*. SN, spontaneous nodule. Asterisks indicate statistically significant differences compared with control (RIN): ***P<0.001; **P<0.01; *P<0.05. Error bars indicate the 95% confidence interval of three biological repeats.

**Fig. 8.**
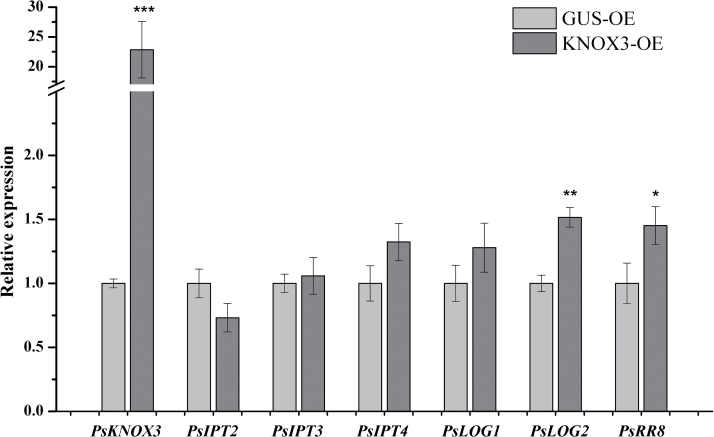
Effect of *KNOX3* ectopic expression on symbiosis-inducible *IPT* and *LOG* genes in transgenic roots of *P. sativum* L. Asterisks indicate statistically significant differences compared with control [*GUS*-overexpressing (GUS-OE)]: ***P<0.001; **P<0.01; *P<0.05. Error bars indicate the 95% confidence interval of five biological repeats.

The expression was determined of symbiosis-inducible *IPT* and *LOG* genes in nodule-like structures formed on *MtKNOX3*-and *PsKNOX3*-overexpressing roots (Figs 7 and 8). As a control, the rhizobium-induced nodules on GUS-overexpressing roots were used. It was found that the expression of *MtLOG2* and *MtIPT3* was higher in nodule-like structures in comparison with the control (*P*<0.001 and *P* <0.01, respectively), while the expression of other analysed genes (*MtLOG1*, *MtLOG3*, and *MtIPT1*) did not show any statistically significant difference (Fig. 7). Likewise, it was found that expression of the *PsLOG2* gene was significantly increased (*P* <0.01) in nodule-like structures on *PsKNOX3*-overexpressing roots (Fig. 8). Moreover, nodule-like structures on *PsKNOX3*-overexpressing roots demonstrated the increased level of cytokinin-responsive *PsRR8* transcripts (*P*<0.05). However, the expression levels of *PsLOG1*, *PsIPT3*, and *PsIPT4* were not significantly increased in the *PsKNOX3* overexpression background (Fig. 8).

### Effect of RNAi on nodule development and the expression of cytokinin biosynthesis/response genes

An RNAi construct was generated (*KNOX3i*) targeting *MtKNOX3* mRNA (see Materials and methods). In *KNOX3i* transgenic roots and nodules, expression of *MtKNOX3* gene decreased 4- to 5-fold compared with control roots (*GUS*-overexpressing control). No statistically significant difference was revealed between nodule number on *MtKNOX3* RNAi roots and control roots (*GUS*-overexpressing roots) (Supplementary Fig. S5, available at *JXB* online). Moreover, nodules on *MtKNOX3* RNAi roots demonstrated the wild-type phenotype (data not shown). The expression levels of other *MtKNOX* genes were not significantly changed by *MtKNOX3* RNAi (data not shown).

**Fig. 9.**
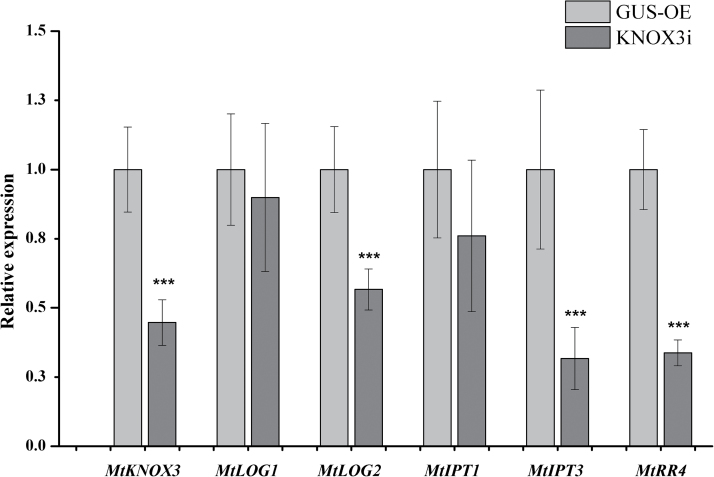
Reduction in gene expression level of *MtLOG2*, *MtRR4*, and *MtIPT3* in *MtKNOX3* RNAi transgenic *M. truncatula* plants. Asterisks indicate statistically significant differences compared with control [*GUS*-overexpressing (GUS-OE)]: *** *P*<0.001, ** *P*<0.01, * *P*<0.05. Error bars indicate the 95% confidence interval of eight biological repeats.

Next, the effect of *MtKNOX3* RNAi on the expression of *MtIPTs*, *MtLOGs* and cytokinin-responsive gene *MtRR4* in transgenic *MtKNOX3* RNAi nodules was analysed with qRT-PCR. A statistically significant decrease was observed in *MtLOG2*, *MtIPT3*, and *MtRR4* gene expression levels in transgenic nodules with *MtKNOX3* RNAi in comparison with *GUS*-overexpressing control nodules (Fig. 9). The expression levels of *Medtr1g110590* (*MtIPT1*) and *MtLOG1* were not significantly changed in the *MtKNOX3* RNAi background (Fig. 9). These data suggested that the MtKNOX3 TF may be involved in the activation of *MtLOG2* and *MtIPT3* genes. The decreased levels of cytokinin-responsive *MtRR4* transcripts in *MtKNOX3* RNAi roots are in line with the suggestion that MtKNOX3 may activate *MtIPT* and *MtLOG* genes and thereby increase cytokinin signalling during nodule development.

## Discussion

Here, it was shown that the expression levels of the *MtKNOX3* and *PsKNOX3* genes encoding class II KNOTTED-like TFs increased significantly during nodulation in *Medicago* and pea. MtKNOX3 was shown to be closest homologue of the KNAT3 protein of *Arabidopsis thaliana* (Di Giacomo *et al.*, 2008). The function of the AtKNAT3 protein is not well characterized. It was reported that *AtKNAT3* is involved in embryo sac formation, germination, and early seedling development in *Arabidopsis*, where AtKNAT3 modulated the abscisic acid response via direct binding and activating the *ABI3* promoter (Pagnussat *et al.*, 2007; Kim *et al.*, 2013). In addition, it was found that *AtKNAT3* expression is repressed by moderate levels of cytokinin (Truernit *et al.*, 2006). The *KNAT3*-like homeobox gene was also studied in black walnut (*Juglans nigra* L.), where it is expressed in pith meristem, roots, embryogenic callus, vascular cambium, leaves, and flowers. This gene is supposed to have a role during heartwood formation (Huang *et al.*, 2009). The broad pattern of *KNAT3*-like gene expression in plants indicates that these genes may have diverse functions and mechanisms of action in plant development.

In this study, promoter:*GUS* analysis showed the *KNOX3* expression pattern in *M. truncatula* nodule primordium cells and in the apical part of the nodule where the meristem is formed, as well as in provascular bundle tissues of developing nodules. *KNOX3* promoter activity was also found in the lateral root primordium, in contrast to its putative orthologue in *Arabisopsis AtKNAT3*, for which expression was not observed in the lateral root primordium. Moreover, Di Giacomo *et al.* (2008) found that *MtKNOX3* expression level increased rapidly in response to exogenous cytokinin by 30min after treatment. This also indicates a difference between *MtKNOX3* and *AtKNAT3*, the latter being shown to be repressed by cytokinin in roots.

Activation of *KNOX3* expression in response to cytokinin indicates a possible link between *KNOX3* and cytokinin action during nodule development. In this study, it was hypothesized that KNOX3 may participate in the activation of cytokinin biosynthesis genes, as was shown for class I KNOX TFs in the SAM (Jasinski *et al.*, 2005; Yanai *et al.*, 2005).

According to the data, the *MtKNOX3* expression maximum at 12 dpi in *Medicago* was associated with the cytokinin response gene *MtRR4* maximum during nodulation. Similarly, the maximum of *PsKNOX3* expression coincided with the expression maximum of the cytokinin response gene *PsRR8*. Moreover, the localization of *KNOX3* expression visualized with promoter:*GUS* analysis coincided with the *MtRR4* expression area in developing nodules studied using promoter:*GUS* analysis and *RNA in situ* hybridization (Vernié *et al.*, 2008; Plet *et al.*, 2011). In the current study, it was found that ectopic expression of the *MtKNOX3* and *PsKNOX3* genes resulted in the formation of bumps exhibiting increased expression of the cytokinin response genes *MtRR4* and *PsRR8*, respectively. The presence of *NIN* gene expression in such structures suggested that they have a nodule-like nature. These data are in line with the hypothesis suggested in this study about the possible role of the *KNOX3* gene in activation of cytokinin biosynthesis genes.

The formation of structures resembling nodule primordia on *KNOX3*-overexpressing roots in the absence of rhizobia inoculation (i.e. spontaneously) may indicate that the TF KNOX3 is involved in the regulation of cell proliferation upon nodule development. It was shown previously that a local increase in cytokinin concentration in *Arabidopsis* root pericycle cells due to overexpression of a cytokinin biosynthesis gene, *IPT*, affected lateral root development. Moreover, the addition of exogenous cytokinin altered the cell division plane of lateral root primordia, disrupting the structure and leading to the formation of ‘flat’ primordia (Laplaze *et al.*, 2007). Lastly, the gain-of function mutation in cytokinin receptor gene *LHK1* in *L. japonicus* (*spontaneous nodule formation 2*, *snf2*) resulted in nodule formation in the absence of rhizobia (spontaneous nodulation) (Tirichine *et al.*, 2007). These data indicate that the increased cytokinin concentration/response in root tissues results in increased cell proliferation of root cortical cells leading to the formation of spontaneous nodules. Thus, here it was assumed that the nodule-like structures observed might also be associated with cytokinin activity, i.e. with the accumulation of active cytokinins in *KNOX3*-overexpressing roots. In contrast to rhizobium-induced nodules, nodule-like structures on *KNOX3*-overexpressing roots developed one central vascular bundle. Previously, such nodule-like structures with one central bundle were observed in transgenic roots with ectopic expression of the *NIN* gene and *NF-Y* subunit genes in *L. japonicus*, which act in the regulatory cascade induced by Nod factors (Soyano *et al.*, 2013). Moreover, nodules with one central vascular bundle are formed in *lin* (*lumpy infection*) mutants defective for rhizobial infection (Kuppusamy *et al.*, 2004; Guan *et al.*, 2013). Xiao *et al.* (2014) suggested that such a phenotype could be explained by the higher cell proliferation in endodermis and pericycle cell layers. Thus, here it was assumed that *KNOX3* ectopic expression changes the phytohormonal balance, which finally induces cell proliferation in the root pericycle, endodermis, and cortex, leading to nodule-like structure formation.

Previously, Chen *et al.* (2013) identified six *IPT* genes in the *L. japonicus* genome and found that *LjIPT1* and *LjIPT3* were activated in response to nodulation. In the current study, it was found that the expression level of *MtIPT1* (*Medtr1g110590*) and its pea homologue, *PsIPT4*, were significantly increased upon nodulation. Changed expression levels were also observed for the *MtIPT3* (*Medtr1g072540*) and *PsIPT3* genes upon inoculation but were not as pronounced as for the *MtIPT1* and *PsIPT4* genes. These data are in agreement with the results obtained for *L. japonicus* by Chen *et al.* (2013). The expression level of *MtIPT4* (*Medtr2g022140*), which was shown previously to be induced early by Nod factor treatment (van Zeijl *et al.*, 2015), was not changed during nodule development in our experiment. It is suggested that the induction of this gene might be associated with the early steps of symbiosis establishment only, since it was induced by Nod factor as early as in 1h after treatment, whereas the later stages of nodule development in our experiment (1 dpi and later in *Medicago*) are unlikely to be regulated by the *MtIPT4* (*Medtr2g022140*) gene. In agreement with this, in pea, induction of the *PsIPT2* gene [*MtIPT4* (*Medtr2g022140*) homologue] was also observed at the early stages of symbiosis development (1–3 dpi). This later response in gene expression following rhizobial inoculation in pea compared with *Medicago* is in line with previous observations that expression of early nodulin genes in pea reached a maximum in between 24 and 48h after rhizobial inoculation (Albrecht *et al.*, 1998; Dolgikh *et al.*, 2011).

The results of this study suggest that the *MtIPT1* (*Medtr 1g110590*)/*PsIPT4* gene is unlikely to be the target of the KNOX3 TF upon nodulation. No significant change in *MtIPT1* (*Medtr 1g110590*)*/PsIPT4* was found in the *KNOX3*-overexpressing background for both legumes. Again, *MtIPT1* (*Medtr1g110590*) expression was not changed in transgenic nodules with *KNOX3* RNAi in *M. truncatula*. At the same time, these data suggest that the *MtIPT3* (*Medtr1g072540*) gene may be regulated by MtKNOX3 during nodulation, since its expression level was decreased in transgenic roots with *MtKNOX3* RNAi and increased in the *KNOX3*-overexpressing background. The homologue of *MtIPT3* (*Medtr1g072540*) in pea, *PsIPT3*, was not significantly upregulated in *PsKNOX3*-overexpressing nodules. Moreover, the *PsIPT3* gene demonstrated a moderate increase throughout the nodule development stages in pea, and one may propose that the role and regulation of the *IPT3* gene during nodule development could differ in both legumes; however, the additional analysis is needed to determine this.

**Fig. 10.**
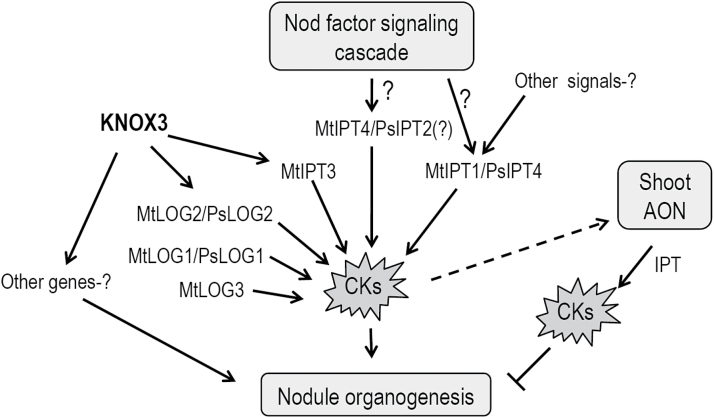
Model of KNOX3 and cytokinin biosynthesis gene action in nodulation. The expression of three *IPT* genes (*MtIPT1/PsIPT4*, *MtIPT3*, *and MtIPT4/PsIPT2*) and three *LOG* genes (*MtLOG1/PsLOG1*, *MtLOG2/PsLOG2*, and *MtLOG3*) were induced in response to *Rhizobium* inoculation. The *MtIPT4* gene (and presumably its pea orthologue *PsIPT2*) may be activated by the Nod factor signalling cascade at the early stages of nodulation, as shown by van Zeijl *et al.* (2015). It is proposed that the *MtIPT3* and *MtLOG2/PsLOG2* genes may be regulated by KNOX3 during nodulation since their expression levels were changed in roots with KNOX3-overexpression and RNAi. This may account for the increase in active cytokinin concentration and, as a consequence, the formation of nodule-like structures on *KNOX3*-overexpressing roots, which demonstrated the increased cytokinin response. Besides its positive effect on nodule formation, cytokinin also acts as negative regulator of nodulation, and its production is induced in shoots by the components of the AON system (Sasaki *et al.*, 2014). CKs, cytokinins.

Lastly, our data suggest that the *LOG2* gene may be regulated by KNOX3 during nodulation. This assumption is supported by the reduced level of *MtLOG2* expression in transgenic roots with *MtKNOX3* RNAi and the increased transcription level of the *MtLOG2* and *PsLOG2* genes in nodule-like structures with *MtKNOX3* and *PsKNOX3* overexpression. Based on these data, it is suggested that KNOX3 may activate *LOG2* gene expression during nodulation, thereby stimulating the accumulation of cytokinin active forms (Fig. 10). This may account for the formation of nodule-like structures on *KNOX3*-overexpressing roots that demonstrated the increased cytokinin response. However, to prove the involvement of the TF KNOX3 in the activation of *LOG2* gene expression upon nodule development, additional experiments are required, such as the measurement of active cytokinin content in transgenic roots with altered expression of *KNOX3* and the identification of KNOX3 direct targets by promoter-binding studies.

In plants, the functions of KNOX TFs are not limited to their effects on cytokinin metabolism genes. These transcriptional regulators are known to regulate the phytohormonal balance via multiple targets, including genes involved in metabolism and action of other hormones such as auxin, gibberellic acid, and abscisic acid (Sakamoto *et al.*, 2001; Bolduc *et al.*, 2012). It is quite possible that KNOX3 also affects multiple gene targets regulating hormonal balance in developing nodules, and future studies are required to unravel KNOX3 targets during nodulation.

## Supplementary data

Supplementary data are available at *JXB* online.

Supplementary Fig. S1. Relative expression of *MtKNOX* genes in uninoculated plants (NI) and at different days post-inoculation (dpi).

Supplementary Fig. S2. Relative expression of *PsKNOX* genes in uninoculated plants (NI) and at different days post-inoculation (dpi).

Supplementary Fig. S3. Tissue-specific expression pattern of pMtKNOX3:GUS in root tips and lateral root primordia.

Supplementary Fig. S4. Phylogenetic tree of the *MtIPT*, *PsIPT*, and *LjIPT* gene families.

Supplementary Fig. S5. Effect of KNOX3 knockdown on nodulation.

Supplementary Table S1. List of primers used for cloning.

Supplementary Table S2. List of primers for qRT-PCR.

Supplementary Data
