# Supplementary material for: KNOTTED1-LIKE HOMEOBOX 3: a new regulator of symbiotic nodule development
Source: J Exp Bot. 2015 Sep 7;66(22):7181–95. doi: 10.1093/jxb/erv414 (PMC4765789; doi:10.1093/jxb/erv414)
Supplement: Supplementary Data [file supp_66_22_7181__index.html]

KNOTTED1-LIKE HOMEOBOX 3: a new regulator of symbiotic nodule development — Supplementary Data 

# KNOTTED1-LIKE HOMEOBOX 3: a new regulator of symbiotic nodule development

## Supplementary Data

Data files

- Supplementary Data - Supplementary Data
