# Supplementary material for: KNOTTED1-LIKE HOMEOBOX 3: a new regulator of symbiotic nodule development
Source: J Exp Bot. 2015 Sep 7;66(22):7181–95. doi: 10.1093/jxb/erv414 (PMC4765789; doi:10.1093/jxb/erv414)
Supplement: Supplementary Data [file supp_erv414_supplementary_corrected_final.pdf]

Figure S1

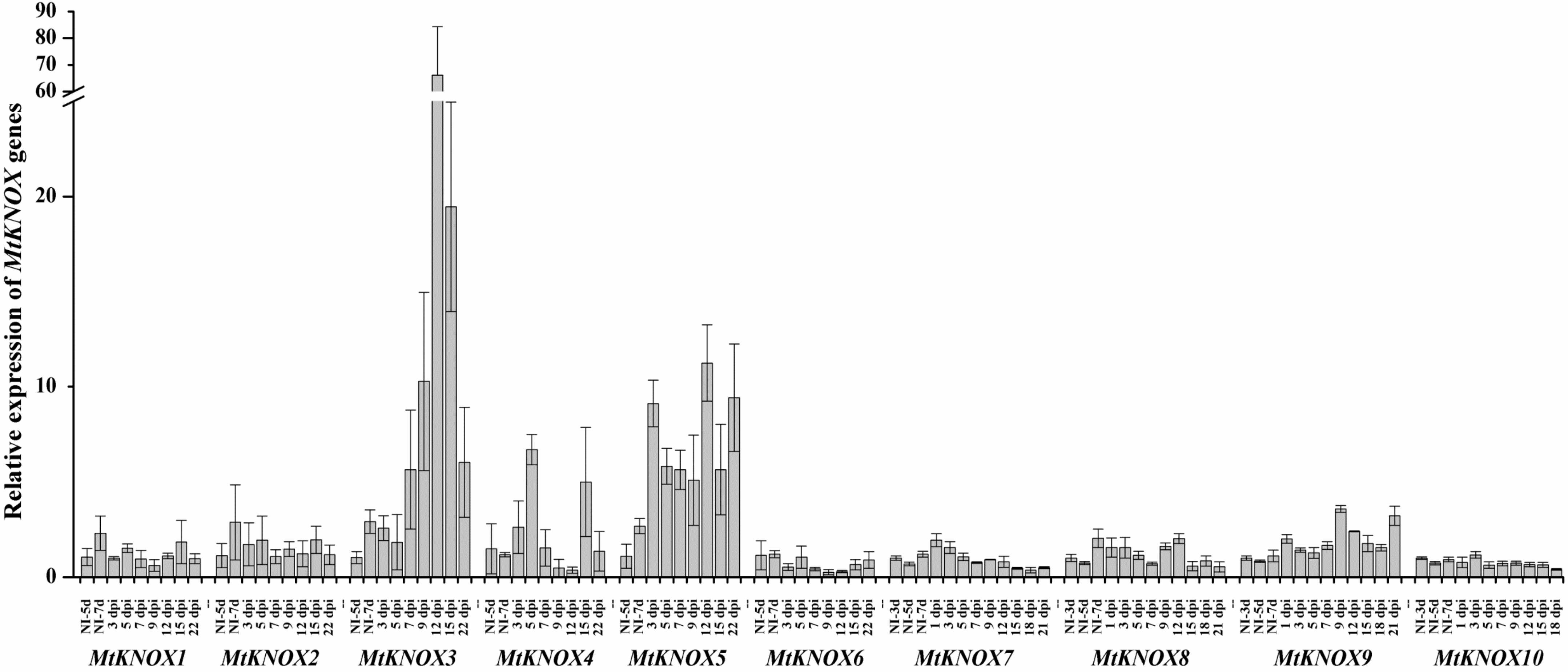

Relative expression of MtKNOX genes
90
80
70
60
20
10
0
MtKNOX1
MtKNOX2
MtKNOX3
MtKNOX4
MtKNOX5
MtKNOX6
MtKNOX7
MtKNOX8
MtKNOX9
MtKNOX10

Figure S2

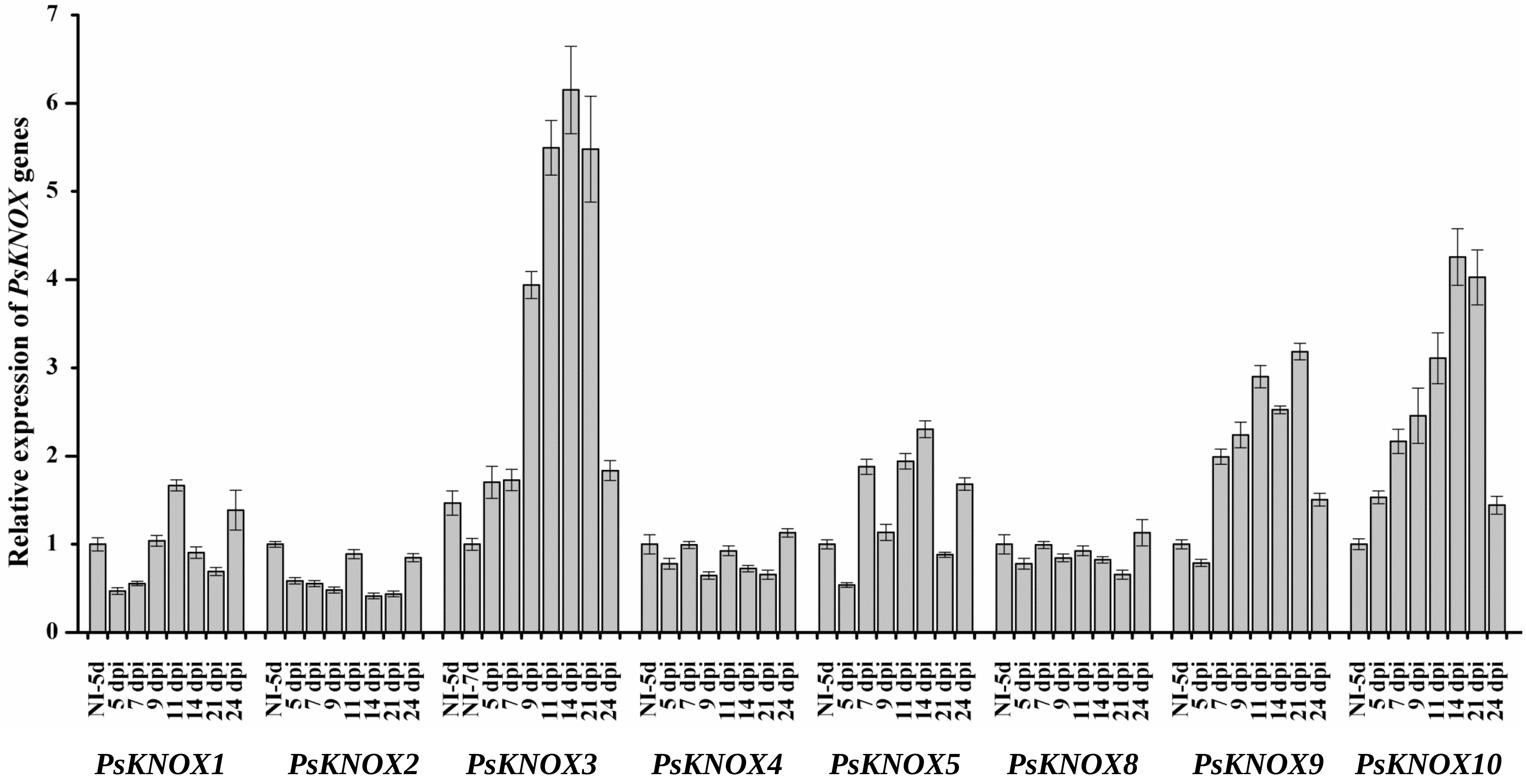
Relative expression of PsKNOX genes
0
1
2
3
4
5
6
7
NI-5d
5 dpi
7 dpi
9 dpi
11 dpi
14 dpi
21 dpi
24 dpi
NI-7d
PsKNOX1
PsKNOX2
PsKNOX3
PsKNOX4
PsKNOX5
PsKNOX8
PsKNOX9
PsKNOX10

Figure S3

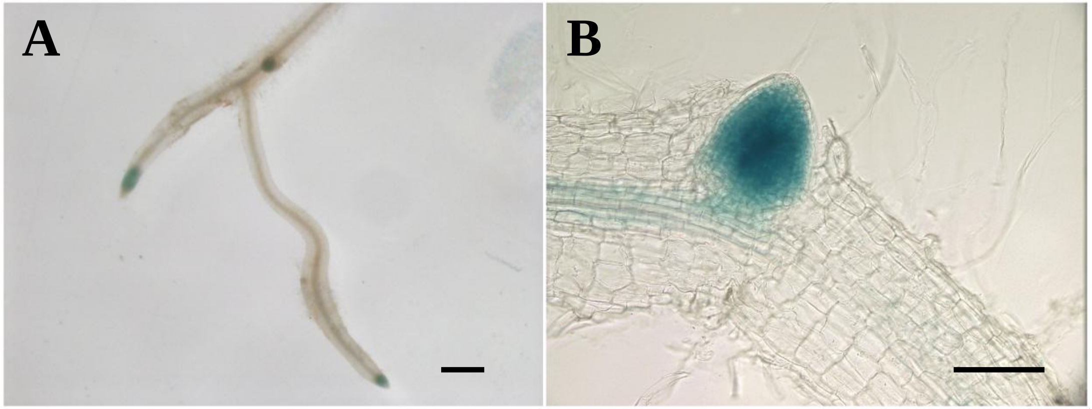

Figure S4

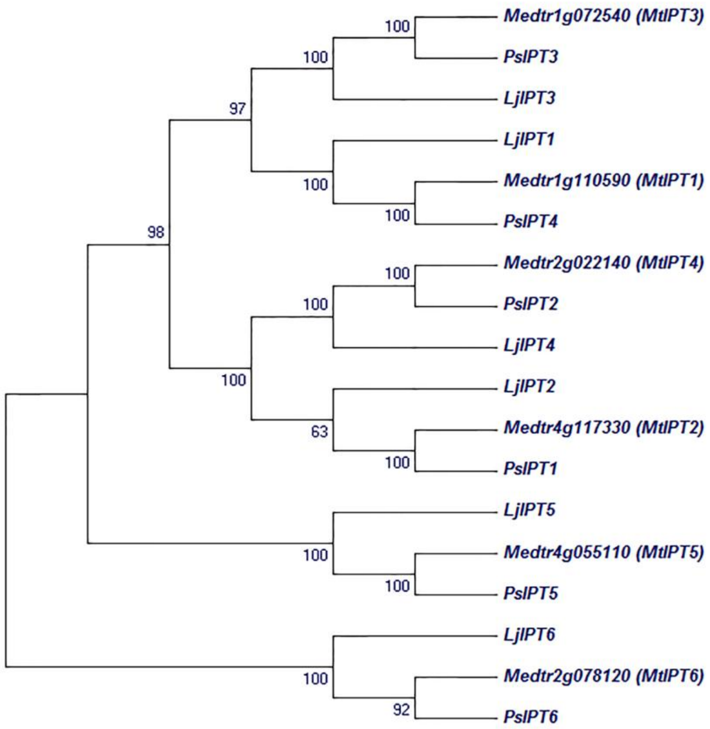

Medicago truncatula	***Lotus japonicus***	**Similarity (%)**
Medtr1g110590 (*MtIPT1*)	*LjIPT1*	67%
Medtr4g117330 (*MtIPT2*)	*LjIPT2*	64,3%
Medtr1g072540 (*MtIPT3*)	*LjIPT3*	71,8%
Medtr2g022140 (*MtIPT4*)	*LjIPT4*	70%
Medtr4g055110 (*MtIPT5*)	*LjIPT5*	80,4%
Medtr2g078120 (*MtIPT6*)	*LjIPT6*	85,3%

Medicago truncatula	***Pisum sativum* L.**	**Similarity (%)**
Medtr1g110590 (*MtIPT1*)	*PsIPT4*	79,6%
Medtr4g117330 (*MtIPT2*)	*PsIPT1*	82,2%
Medtr1g072540 (*MtIPT3*)	*PsIPT3*	84,4%
Medtr2g022140 (*MtIPT4*)	*PsIPT2*	85,5%
Medtr4g055110 (*MtIPT5*)	*PsIPT5*	84,7%
Medtr2g078120 (*MtIPT6*)	*PsIPT6* (CDS partial)	90,9%

Figure S5

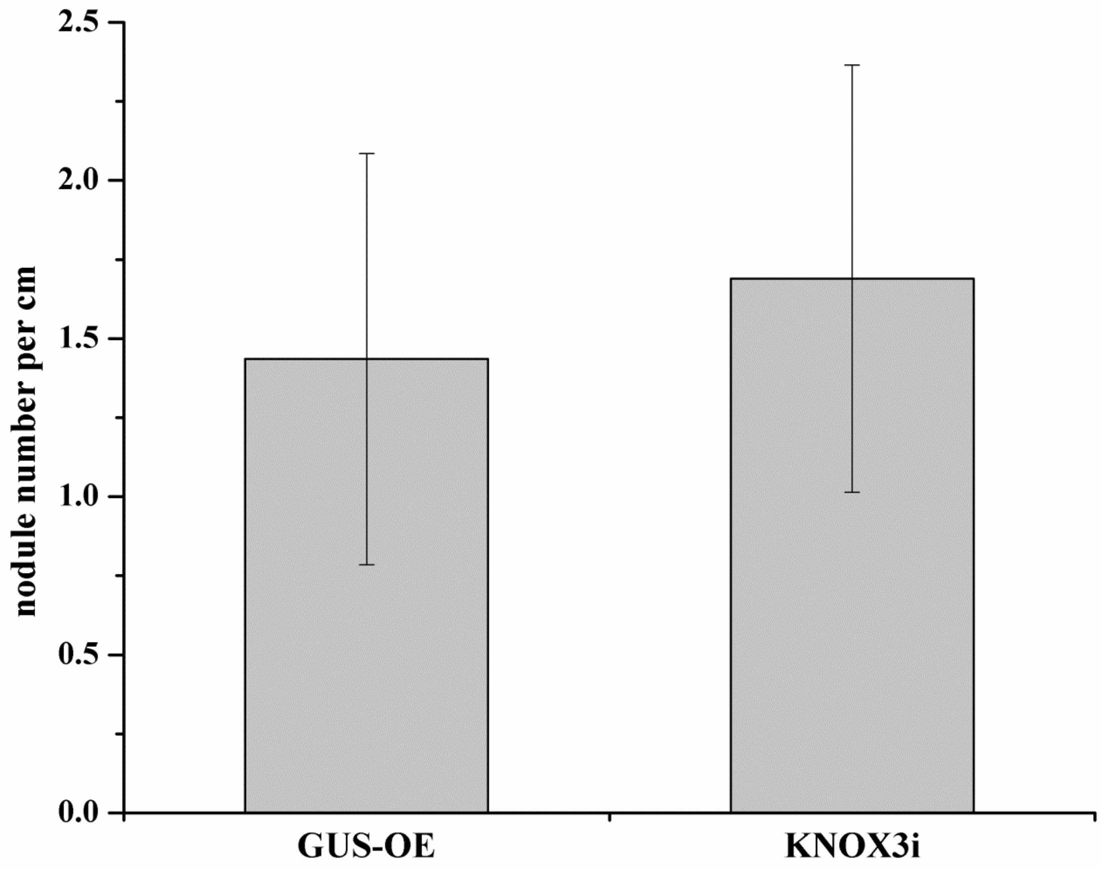

Table S1.

List of primers used for cloning.

gene	Forward primer	Reverse primer
MtKNOX3-OE	5'-AAAAAAGCAGGCTTCATGGCTTACCAAAACCAACATCT-3'	5'-CAAGAAAGCTGGGTTCTAGTTTTGAGACCTTTTGCGTTT-3'
MtKNOX3i	5'-CACCATGGCTTACCAAAACCAACATC-3'	5'-TTGGTTATTAGGGTCGGAATCA-3'
PsKNOX3-OE	5'- GGAGATAGAACCATGGCTTAT CACAACCAGCATC-3'	5'- AAGCTGGGTGCTAGTTTTGAG ACCTTTTGCGC-3'
attB_primers	5'-GGGGACAAGTTTGTACAAAAA AGCAGGCTTC-3'	5'-GGGGACCACTTTGTACAAGAA AGCTGGGTG-3'
*PsKNOX3*_check	5'-CTCCTTTGTTCAAACACATAA-3'	5'-TTAGTGCAATTATCATCCCT-3'
PsKNOX3_3'_F1_ RACE (cv. Frisson)	5'-CAAAGCTTAACAGGAGTTTCACC-3'	
PsKNOX3_3'_F2_ RACE (cv. Frisson)	5'-GATTTGGCCCTCTTATACCTACAG-3'	
PsKNOX3_3'_F3_ RACE (cv. Frisson)	5'-AAGAGACGAGCCGGGAAACT-3'	
PsKNOX3_5'_R1_ RACE (cv. Frisson)		5'-AGTTTCCCGGCTCGTCTCTT-3'
PsKNOX3_5'_R2_ RACE (cv. Frisson)		5'-CTGTAGGTATAAGAGGGCCAAATC-3'
PsKNOX3_5'_R3_ RACE (cv. Frisson)		5'-GGTGAAACTCCTGTTAAGCTTTG-3'
*PsKNOX4*_check	5'-ACCTCAACCCTCTTTCCT-3'	5'-AGTACTCTATCATCAATAACCATA-3'
*PsKNOX5*_check	5'-ACTCTCAACTTCTCCTTCGC-3'	5'-CATATATATGTAATCACCATCAC-3'
PsKNOX6-3'_F1 RACE	5'-ATGGCTCATCCTCTCTTTCC-3'	
PsKNOX6-3'_F2 RACE	5'-ATGTGAATTATTGAATGG-3'	
PsKNOX6-3'_F3 RACE	5'- GTTTGGATCAGTTCATGGAG -3'	
PsKNOX6-5'_R1 RACE		5'-CTCCATGAACTGATCCAAAC-3'
PsKNOX6-5'_R2 RACE		5'-CCATTCAATAATTCACAT -3'

PsKNOX6-5'_R3 RACE		5'-GGAAAGAGAGGATGAGCCAT-3'
PsKNOX8_3'_F1_ RACE	5'-TTGAATGAAGCGAGTTTGTTTTT-3'	
PsKNOX8_3'_F2_ RACE	5'-CAAATGCTCCTTCGTAAATATGG-3'	
PsKNOX8_3'_F3_ RACE	5'-GAGAAAATGCAGCTATCAGAAACA-3'	
PsKNOX8_5'_R1_ RACE		5'-CCATATTTACGAAGGAGCATTTG-3'
PsKNOX8_5'_R2_ RACE		5'-TTCAACTGCTTCAACCTTTCCACA-3'
PsKNOX8_5'_R3_ RACE		5'-GGAGACTATCGGTGCGGTGG-3'
*PSKNOX9*_check	5'-ATGCAGTGAGAGAAGAGAAAG-3'	5'-GATAACATGAAATGAAGAAG-3'
*PSKNOX10*_check	5'-CGAATCAAATTTCGCAGTAGA-3'	5'-ATATTAATAATTCTTTCTTTCATCA-3'

Table S2

List of primers for qRT-PCR.

Gene	Gene ID/ GenBank accessions numbers	Forward primer	Reverse primer
Ubi	Medtr4g091580 *(M. truncatula Polyubiquitin 3)*<br>L81140.1 (*P. sativum polyubiquitin 2 (PUB2*)	5’- ATGCAGATYTTTGTGAAGAC -3’	5’- ACCACCACGRAGACGGAG -3’
*MtACTIN11**	Medtr7g026230	5’-ACGAGCGTTTCAGATG-3’	5’-ACCTCCGATCCAGACA-3’
*MtKNOX1***	EF128056<br>Medtr2g024390	5’-ATTCATTCAATGGAGGGTAGTT-3’	5’-TTTTGAAAGCATGATAGAAGAGGT-3’
*MtKNOX2***	EF128057<br>Medtr1g017080	5’-GAAGCGTTGACACTGGCACATC-3’	5’-TGGAAAATAGAAGCAACAGACACT-3’
*MtKNOX3***	EF128058<br>Medtr1g012960	5’-TGCACCAAACTCCCCTAAGAT-3’	5’- TTCCTAAAACAAAAAGACTAA-3’
*MtKNOX4***	EF128059<br>Medtr5g011070	5’-AGTGTACTGTAGACCCTCCAATG-3’	5’-ACCACACCTAATAATAACAACAGA-3’
*MtKNOX5***	EF128060<br>Medtr3g106400	5’-GGTTCCCAATCCTCCGGTCAATGG-3’	5’-TCGTTATCAAGCATGCCCT-3’
*MtKNOX6***	EF128061<br>Medtr5g085860	5’- CATGTGCAAGCCATTCCCTATG-3’	5’- GAAAAATCAACCGTTATGCCCA-3’
MtKNOX7	XM_003613135<br>Medtr5g033720	5’-AACTGCTTCTGATGATGGTGGTG-3’	5’-GGGTTTGTCTTGCTTCTTTTGGA-3’
MtKNOX8	XM_003591173<br>Medtr1g084060	5’-CGCAACTCACCCTCTTTATCCA-3’	5’-GCTTTCACGCCCTATTTCTTCA-3’
MtKNOX9	Medtr4g116545-00360952	5’-TTATGTTCTTTTGCTGTGTGCCTTC-3’	5’-CTCTCTGCCTGCTCGTCTTCA-3’
MtKNOX10	Medtr2g461240	5’-TTGCTAATGCTAATGGTGTTGTGG-3’	5’-CTCTGCTTGGTCATCTTCATCGT-3’

PsActin	X67666.1	5’-CTCAGCACCTTCCAGCAGATGTG-3’	5’-CTTCTTATCCATGGCAACATAGTTC-3’
PsKNOX1	AF080104.2	5’-TTACTTGAATGGTGGAGCAGACA- 3’	5’-ATGGGAAATGAATTGGTGAGAAC-3’
PsKNOX2	AF080105.1	5’-ATCAACATCATCATCATCAACAAC-3’	5’-GTCTAGTTCTGGATCTTTTGAAGA-3’
PsKNOX3	KP296686/ KP296687	5’- ATTCCGATCCCAACCAACTCC-3’	5’-ACTTACTCCTCCGTCGCCTTTCAA-3’
PsKNOX4	KP296688	5’-CAACTCAAGGCGGAGATAGCAA-3’	5’-ATATCACGGCAAGCCATAACA-3’
PsKNOX5	KP296689/ KP296690	5’-ACCTCCGAACCTCACCCATCCT-3’	5’-TGCGCCATTATTTCCCCTTTGTAT-3’
PsKNOX6	partial CDS	5’-ATGGCTCATCCTCTCTTTCC-3’	5’-CTCCATGAACTGATCCAAAC-3’
PsKNOX8	KT363851	5’-TTGAATGAAGCGAGTTTGTTTTT-3’	5’-CCATATTTACGAAGGAGCATTTG-3’
PsKNOX9	KP296691	5’-CACTTGGAACCGGAGAGGAGAG-3’	5’-GAGTTGCGATTCGGAGACACG-3’
PsKNOX10	KP296692	5’-ACATGCAAAGGCTACTACCGTTCA-3’	5’-AATTCCTCTTCCGTCGTCGTTTTG-3’
MtIPT1	Medtr1g110590	5’-TCGCCACCCTCTTCCCTTAC-3’	5’-CCGACAACACAGGAAACGAAA-3’
MtIPT3	Medtr1g072540	5’-TCCTCCAAATCACCAAACATAAAA-3’	5’-CGCCCATCACCAACACTACA-3’
MtIPT4	Medtr2g022140	5’-GCCACCGAAACGCACCTTA-3’	5’-ATTCCCTGTCCCACCACCTC-3’
PsIPT2	AB194605.1	5’-GGAAAGACCAAATTAGCCATAGAT-3’	5’-AACCCAAAGAAAACAACACTCATA-3’
PsIPT3	KP296693	5’- AATCACGGGCCACGGACATC-3’	5’- CATCGACCCAAAGACAACAGAAG-3’
PsIPT4	KP296694	5’-ATCCCCTTTCATCAACGCAACAA-3’	5’- GTCCACCATTCCCGAGTCAAACAT-3’
MtLOG1	Medtr7g101290.1	5’-GGGGATATGGAACTCTGGAGGA-3’	5’-TGTCATCGCAACAACGGAAA-3’
MtLOG2	Medtr1g064260.1	5’-TAACTGGTGAAACGGTGGGAGA-3’	5’-CCAACTCTTTTGATGTAGGTGCTGA-3’

MtLOG3	Medtr4g058740.1	5’-ATTGGTTTGATGGGGTTGGTTT-3’	5’-AGCTCTTTTGTTGATGGTGCAGA-3’
MtLOG4	Medtr3g113710.1	5’-ACGCATCTGTGTTTATTGTGGTAGC-3’	5’-GTTCTTCGAGGGTGCCATATCC-3’
MtLOG5	Medtr1g105240.1	5’-TCAACGCCTCTTTTCTCCATCA-3’	5’-CAGACCCACACTACCACCTCCA-3’
MtLOG6	Medtr1g015830.1	5’-GGTGGAAGTGTTGGATTGATGG-3’	5’-AAGTTCTTTGGCTGATGAAGCTGA-3’
LOG_Medtr3g041540	*LOG_Medtr3g041540*	5’-GGTTTCCATTTTTGCGTTGAGG-3’	5’-TGACCTTTTCACAGTCCATTCGT-3'
PsLOG1	KP296697	5’-TGACAAGCCGGTGGGATTAGTAG-3’	5’-ATTATGTGACGAGCATTTGGACTG-3’
PsLOG2	KP296698	5’-ACAAGCTGTTCATGATGGTGGTAGA-3’	5’-AGCTTTGTCAATGAATGACAGTAAC-3’
*MtRR4**	Medtr5g036480	5’-ATGCTTTTGTTCCGGGTTTA-3’	5’-CTGCACCTTCCTCCAAACAT-3’
PsRR4	KP296699	5’-GGAGGAAGGTGCAGAGGATTTTA-3’	5’-GGGCGAAGAAGACAAAGGAGATG-3’
PsRR8	KP296702, KP296703	5’-TTGAGGAAGGAGCGGAGGAT-3’	5’-AATGTGGCGGATAGAGATGGGC- 3’

* Sequence of primers for *MtActin11* and for *MtRR4* were taken from Gonzalez-Rizzo et al., 2006

**Sequence of primers for *MtKNOX1-6* were taken from Giacomo et al., 2008
